# Proton-Induced X-ray Emission (PIXE) Analysis and DNA-chain Break study in rat hepatocarcinogenesis: A possible chemopreventive role by combined supplementation of vanadium and beta-carotene

**DOI:** 10.1186/1475-2867-5-16

**Published:** 2005-05-26

**Authors:** Mitali Basu Chattopadhyay, Sutapa Mukherjee, Indira Kulkarni, V Vijayan, Manika Doloi, NB Kanjilal, Malay Chatterjee

**Affiliations:** 1 Division of Biochemistry, Department of Pharmaceutical Technology Jadavpur University, Kolkata 700 032, India; 2 Institute of Physics, Sachivalaya Marg, Bhubaneswar – 751 005, Orissa, India; 3 All Indian Institute of Hygiene and Public Health, Kolkata 700 072, India

**Keywords:** DENA, vanadium, PIXE, beta-carotene, DNA-chain break

## Abstract

Combined effect of vanadium and beta-carotene on rat liver DNA-chain break and Proton induced X-ray emission (PIXE) analysis was studied during a necrogenic dose (200 mg/kg of body weight) of Diethyl Nitrosamine (DENA) induced rat liver carcinogenesis. Morphological and histopathological changes were observed as an end point biomarker. Supplementation of vanadium (0.5 ppm *ad libitum*) in drinking water and beta-carotene in the basal diet (120 mg/Kg of body weight) were performed four weeks before DENA treatment and continued till the end of the experiment (16 weeks). PIXE analysis revealed the restoration of near normal value of zinc, copper, and iron, which were substantially altered when compared to carcinogen treated groups. Supplementation of both vanadium and beta-carotene four weeks before DENA injection was found to offer significant (64.73%, P < 0.001) protection against generation of single-strand breaks when compared with the carcinogen control counter parts. A significant stabilization of hepatic architecture of the cells was observed as compared to carcinogen control in vanadium plus beta-carotene treated group. This study thus suggests that vanadium, a prooxidant but potential therapeutic agent yield safe and effective pharmacological formulation with beta-carotene, an antioxidant, in the inhibition of experimental rat hepatocarcinogenesis.

## Background

Trace element studies on cancer and normal tissues have interested groups of researchers for many years [1]. Accumulating evidence demonstrated that many essential/trace elements play an important role in a number of biological processes by activating or inhibiting enzymatic reactions, by competing the permeability of cell membranes, maintaining genomic stability and/or by other mechanisms. It is therefore, reasonable to assume that this element would exert action, directly or indirectly on the carcinogenic process [1,2]. The technique of PIXE (Proton induced X-ray emission), since its introduction has been successfully used by various groups throughout the world for trace elemental analysis of biomedical samples due to high sensitivity, multielemental capability and its non-destructive nature [3].

Vanadium (**V**), which is an endogenous constituent of all or most mammalian tissues, is believed to have a regulatory role in biological system [4]. Previous studies from our laboratory [5] and other laboratories [6,7] have established **V **as a significant biological regulator in assessing the physiological and biochemical state of the animals in a dose related manner [6]. It has been shown that **V **at 0.5 ppm in drinking water was effective in inhibiting the chemical induced two stage rat hepatocarcinogenesis without any toxic manifestation [8]. The data from several animal experiments established the role for beta-carotene (BC) as an antioxidant and even as an anticancer agent. [9]. BC has been found to be prophylactic for UV and carcinogen-induced cancers of the skin, mammary gland, buccal pouch epithelia and colon in animal models [10,11]. This current work is directed to elucidate the efficacy of both the trace element **V **and the antioxidant BC when given together can effectively prevent the transformation of neoplastic cells of liver treated with a single necrogenic dose of diethylnitrosamine (DENA).

The use of single injection of DENA allows observation of the chemopreventive potential of **V **and BC in combination in the initiation phase of hepatocarcinogenesis by the DNA chain break analysis. DNA strand can be detected with great sensitivity by methods that utilize observation of the role of the unwinding of the two DNA strand [12]. In this study, we have used a technique of fluorimetric analysis of DNA unwinding (FADU) using a fluorescent dye for monitoring DNA unwinding, according to our method of Sarkar *et al*., 1997 [13]. In addition, a 16-weeks study was further conducted first, to demonstrate the protective response of combined supplementation of **V **and BC in modulating morphological, histopathological changes and second to examine the role of micronutrient concentrations in the carcinogenic process.

Information on the combined effect of vanadium and beta-carotene and their antineoplastic potential is still meager. In this communication, we report for the first time that the trace element, vanadium in combination with antioxidant, beta-carotene can modulate the activity/or status/ or concentration of other elements at the particular dose and tend to stabilizes the genome to some extent in chemical carcinogenesis.

## Materials and methods

### Animals and diet

Male Sprague Dawley rats weighing 80–100 g at the beginning were obtained from the Indian Institute of Chemical Biology (CSIR), Kolkata 32, India and maintained on standard basal diet. We followed the recommendations of the NIH guide for the Care and Use of Laboratory Animals for the maintenance, treatment and sacrifice of the animals used in this study.

### Experimental design

**Figure 1:**
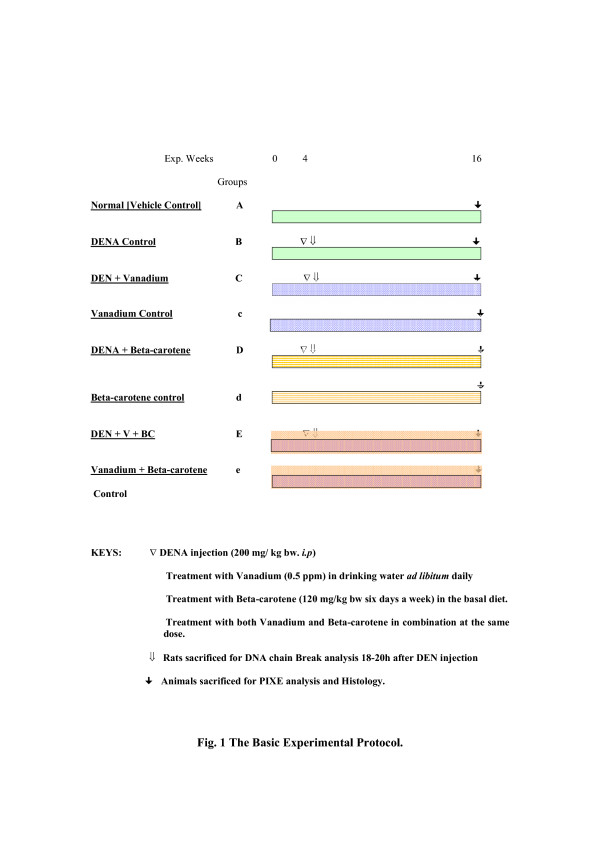
The Basic Experimental Protocol.

Two separate sets of rats were used for the study one for PIXE analysis and histopathological study (Set-I) another for the DNA-Strand assay (Set-II). In Set-I, the rats were divided into eight (8) groups (Fig. 1) with ten rats in each. In group B, C, D and E, hepatocarcinogenesis was initiated by a single intraperitoneal (*i.p*) injection of DENA at a dose of 200 mg/Kg body weight in 0.9% sodium chloride solution [8,15] at week four. Normal vehicle control rats (Group A) received only sodium chloride solution till the end of the experiment (16 weeks). **V **(as ammonium monovanadate) was supplemented at a dose of 0.5 ppm in drinking water *ad libitum *[8] and BC was given in the basal diet at a regimen of 120 mg/Kg of body weight [13] six days a week for the entire period of study. Group B rats were the DENA controls, group C were supplemented with **V **only, group D rats were treated with BC only and group E were treated with both **V **and BC. Group c, d and e served as the respective **V**, BC and **V **plus BC control groups. For DNA-strand breaks analysis (Set-II) rats were supplemented at the same dose regimen of PIXE analysis four weeks prior to DENA injection. All the rats were killed 18–20 h after DENA injection, livers are promptly excised and DNA was isolated.

### Experimental set-up for PIXE analysis

About 5 ml of whole blood samples from different control and experimental groups were collected and lyophilized at the Regional Medical Research Center (RMRC), Bhubaneswar. Then samples were converted into fine powder, homogenized and finally pressed into pellets. Conversion of the sample into fine powder before pelletising is necessary to avoid particle size effects in the analysis. These effects are particularly important for the lighter elements. To obtain consistent pellets which remain stable during bombardment, an equal amount of binder such as graphite are added to the powdered sample prior to pelletisation so that bombardment in vacuum does not lead to charging of specimen [14]. The targets are normally thin layers of the sample deposited uniformly over Mylar films usually of 3 μm to 6 μm thickness. Yttrium is added as internal standard. During each run a set of IAEA (international atomic energy agency) animal blood reference standard A13, are prepared and analyzed along with other biomedical samples under similar experimental conditions.

### Morphology of liver

For histological studies, representative longitudinally cut blocks were taken from the left, right-median and right-anterior lobes and the samples were fixed immediately in 10% buffered formalin to prevent deformation. Five contiguous sections were made from each liver slice for routine histopathological evaluation by hematoxylin and eosin (H & E) staining. Specific hepatocellular lesions observed in H & E staining were recognized by light microscopy (Adcon, Model No. 5591) according to published criteria [15].

### DNA Strand break assay

DNA was isolated from the rat liver by a modification of the published criteria [16]. After isolation of DNA from each experimental and control group, DNA was assayed for extent of DNA unwinding by the FADU technique [13]. Data were analysed statistically or differences between the means using Student's t-test.

### Statistical analysis

Significance of difference between mean values of different groups of rats was assessed by Student's *t*-test values of P < 0.05 were taken to imply statistical significance.

## Result

### Effect of Vanadium and Beta-carotene on essential/Trace elements

Thick target PIXE analysis was performed using GUPIX-95 software, which provides non-linear least squares fitting of the spectrum, together with subsequent conversion of the X-ray peak intensities (not shown) into elemental concentrations via a defined standardization technique involving fundamental parameters and user defined instrument constant. Full account was taken matrix effects and secondary fluorescence contributions in both the spectrum fitting portion and the calculation of concentrations.

Mean concentrations of 8-essential/trace elements were measured by PIXE analysis of blood samples of different groups are shown in Table 1. Results from this study show that the content of elements calcium (Ca), copper (Cu) and zinc (Zn) were lower in the DENA control Group B than the certified values (Table I). On the other hand, the element potassium (K), iron (Fe), manganese (Mn), selenium (Se) showed elevated concentrations in Group B (DENA control). In contrast, elemental analysis of blood samples in group C, D and E treatment with **V **or BC or both in combination exhibited marked closeness to normal value (Table 1). Data for the control groups of animals (group c, d and e) did not show any marked differences from the normal vehicle control (Group A) counterparts and hence were not included in the study.

**Table 1:** The Elemental concentrations in μg/ml of five different groups. The Values given in column 2 are the certified concentrations (reference standard A13) of various elements.

**Elements Detected (in μg/ml)**	**Certified Value**	**A (Normal)**	**B (DENA Control)**	**C (DENA+V)**	**D (DENA+BC)**	**E (DENA+V+BC)**
K	2500 ± 350	3714 ± 0.03^a^	5440.6 ± 0.02^b^	3810.5 ± 0.04^c^	3805.0 ± 0.04^c^	3782.5 ± 0.01^d^
Ca	286 ± 53	269.7 ± 0.07	119.1 ± 0.03^b^	242.5 ± 0.02^c^	247.6 ± 0.02^c^	261.9 ± 0.022^d^
Mn	1.4 ± 1	1.6 ± 0.06	2.0 ± 0.05^b^	1.5 ± 0.023^c^	1.8 ± 0.01^c^	1.6 ± 0.01^d^
Fe	356 ± 150	370.5 ± 0.04	577.5 ± 0.04^b^	392.7 ± 0.03^c^	387.0 ± 0.06^c^	379.8 ± 0.02^d^
Cu	2.3 ± 0.5	1.5 ± 0.06	0.89 ± 0.05^b^	0.98 ± 0.06^c^	0.96 ± 0.02^c^	1.2 ± 0.01^d^
Zn	13.0 ± 10	18.9 ± 0.01	9.0 ± 0.06^e^	17.9 ± 0.02^f^	17.3 ± 0.04^f^	18.4 ± 0.04^g^
Se	0.24 ± 0.5	0.18 ± 0.04	0.19 ± 0.03^b^	0.10 ± 0.07^c^	0.16 ± 0.05^c^	0.18 ± 0.07^d^
Pb	0.18 ± 0.01	0.21 ± 0.03	0.25 ± 0.04^m^	0.22 ± 0.02^n^	0.24 ± 0.03^n^	0.22 ± 0.03^l^

^a^Values are mean ± S.E. of five different run using different sets of targets.

^b^*p *< 0.01 as compared with group A,

^c^*p *< 0.01 as compared with group B,

^*d*^*p *< 0.001 as compared with group B.

^e^*p *< 0.005 as compared with group A,

^*f*^*p *< 0.01 as compared with group B,

^*g*^*p *< 0.005 as compared with group B.

^*m*^*p *< 0.001 as compared with group A,

^n^*p *< 0.05 as compared with group B,

^*l*^*p *< 0.005 as compared with group B.

### Effect of vanadium and beta-carotene on hepatic Histopathology

**Figure 2:**
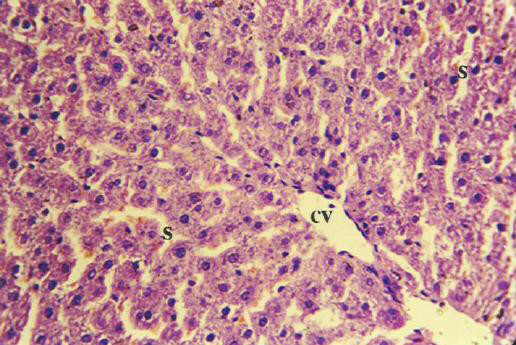
Thin section of the normal untreated rat liver (Group A) showing normal hepatocellular architecture [H&E × 400].

**Figure 3:**
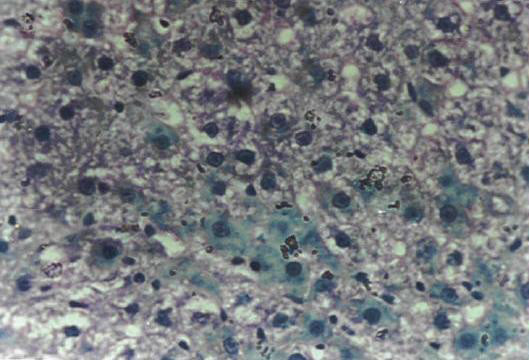
Section of the rat liver (Group B) showing abnormal architecture after initiation with DENA (200 mg/ Kg b. wt.) for 16 weeks [H&E × 400].

**Figure 4:**
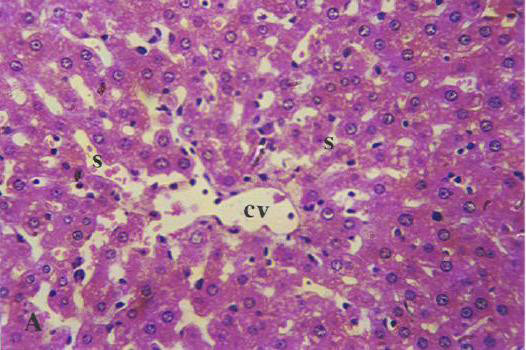
Section of the DENA treated rat liver (Group E) showing almost normal architecture after continuous supplementation of vanadium (0.5 ppm) and beta-carotene (120 mg/kg of basal diet) for 16 weeks. [H&E × 400]. **cv**, central vein;

**Figure 5:**
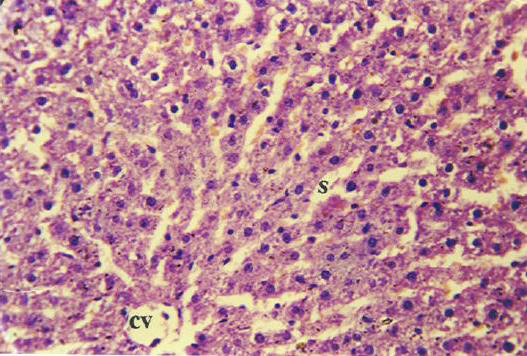
Thin section of the rat liver of vanadium and beta-carotene control (Group e) showing normal hepatocellular architecture, CV, Central Vein [H&E × 400].

Histopathological examination in liver sections from normal untreated group, i.e. Group 'A' (Figure 2) as well as other control groups, i.e. Groups 'c', 'd' (Figures not shown) and 'e' (Figure 5) which received **V **or BC alone or both in combination respectively revealed normal liver parenchymal cells with granulated cytoplasm and small uniform nuclei radially arranged around the central vein. On the contrary, gross structural alterations were seen in Group B rats (DENA control) with predominantly basophilic, eosinophilic and clear cell foci (Figure 3). Extensive vacuolation was observed in the cytoplasm encircling the nucleus with masses of acidophilic materials. Some nuclei in the cells were large and hyperchromatic with prominent and centrally located nucleoli (Figure 3). Phenotypically altered hepatocyte populations in the form of altered liver cell foci and nodules in varying extent were noticeable throughout the hepatic parenchyma of all groups treated with DENA. The cellular architecture of hepatic lobules in Group E rats (Figure 4) those received both **V **and BC in combination for 16 weeks seemed to be almost comparable to normal. This elicited the maximum protection of both **V **and BC (Group E) for the entire period of study against DENA-induced hepatocarcinogenesis, which was reflected in the almost normal hepatocellular architecture. Liver cells from this group were found to contain compact cytoplasmic material with only clear cell foci (Figure 4). The nucleocytoplasmic ratio was decreased considerably as compared to Group B (DENA control). However, a moderate improvement in hepatic cellular architecture was observed in Groups C (Figure not shown) and D (Figure not shown) rats receiving **V **and BC alone respectively. A considerable vacuolation was still observed in the cytoplasm and the compactness of the hepatocytes was not like normal untreated control group (Group A). The liver sections from these groups presented a predominance of clear cell foci rather than eosinophilic and/or basophilic foci.

### Effects of Vanadium and Beta-carotene on DEN-induced hepatic DNA chain break

**Figure 6:**
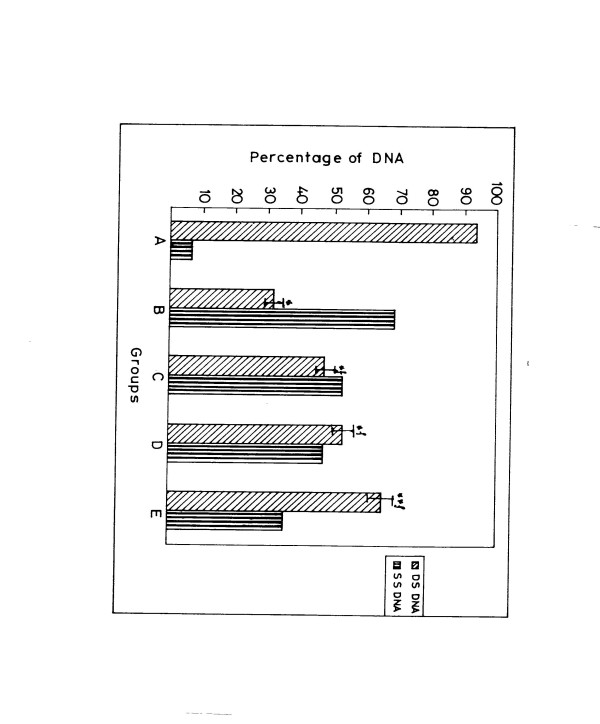
Inhibitory effect of vanadium and beta-carotene (in combination) on the generation of DNA-strand breaks initiate with DENA. *P < 0.01 as compared to Group A *^f^P < 0.01 as compare with Group B. **^f^P < 0.005 as compared with Group B.

A significant rise in total percentage of hepatic DNA single-strand break could be observed in group B rats 20 h following DENA injection (Fig. 6) when compared with normal control, i.e., Group A. The percentage of native double-stranded DNA of group B (DENA control) rats was found to be three-fold less (P < 0.001) whereas the total aberrant single-strand regions were found to be more than ten fold (P < 0.001) higher than that of normal vehicle control (Group A) [Fig. 6]. This is actually shows the direct DNA damaging efficacy of the hepatocarcinogen DENA. Treatment with V or BC or both in combination in group C, D and E rats respectively strictly abated the generation of single-stranded DNA 20 h following DEN injection. Result shows that maximum protective effect against generation of single-stranded region was found in Group E rats that received both V and BC. Moreover, the native double-stranded DNA in group E rats was almost two fold higher than in DENA control rats (Group B) [Fig. 6]. Table 2 shows the number of single-strand breaks/ DNA 20 h following DENA injection in the presence of V and BC either alone or in combination. In group B, a significant (P < 0.01) increase in the number of single-strand breaks/DNA could be observed. Treatment with V or BC alone showed decrement at the level of 47.23% and 52.87% respectively (P < 0.01) in the generation of single-strand DNA over and above DENA control but the maximum protective effect (64.73%, P < 0.005) was found in Group E that received both V and BC in combination.

**Table 2:** Effect of vanadium and beta-Carotene on the number of single-strand breaks/DNA fragment in rat liver 18–20 h after a single injection of DEN

**Group**	***Treatment***	**Percentage of Double-strand DNA**	**Number of single-strand breaks/DNA fragment**	**Inhibition (%)**
A	Normal	93.56	0.07 ± 0.02^a^	
B	DENA Control	31.68	1.32 ± 0.01 ^b^	
C	DENA+V	47.23	0.58 ± 0.01 ^c^	56.06
D	DENA+BC	52.87	0.54 ± 0.04 ^c^	59.09
E	DENA+V+BC	64.73	0.48 ± 0.023 ^d^	63.6

The mean values of the number of the single-strand breaks/DNA fragment for Vanadium control (group c) was 0.07 ± 0.01; for β-carotene control (group d), 0.08 ± 0.01 and the Vanadium and beta-carotene control (group e), 0.10 ± 0.01. No statistical significance could be observed between these drug controls when compared with normal vehicle control (Group A).

^a^Values are means ± S.E of 5 rats (each rat liver genomic was tested in triplicate)

^b^*p *< 0.01 as compared with group A,

^c^*p *< 0.01 as compared with group B,

^*d*^*p *< 0.005 as compared with group B.

## Discussions

The **V **plus BC supplemented group E animals registered a 64.73% inhibition in the number of single strand-breaks per DNA (Fig. 6). Such strand breaks are generally assumed to be lethal to cells. It is well known that DNA strand breaks responsible for chromosomal alterations are generated from DNA base lesions induced by most chemical mutagens. The substantial decrement of the single strand breaks can explain one possible mechanism of the anticlastogenic potential of **V **and BC. One hypothesis is that the *in vivo *protective effect of **V **and BC when give together as observed here, may be due to excision repair activity. DNA double-strand breaks (DDBs) are generated from mutagen induced DNA lesions in the S phase of the cell cycle and repaired by post replication repair in the G2 phase and that unrepaired DDBs result in breakage type chromosomal aberrations [17]. In this context, the suppression of break type aberrations by the combined action of **V **and BC may be due to a modification of the capability of the post-replication repair of DDBs.

Several reports suggest that antioxidants suppress the clastogenic action of tumor promoters and carcinogens [18]. The inhibitory effect of different antioxidants like vitamin E, beta-carotene and selenium in the production of SCEs in cultured mammalian cells by stimulated human phagocytes, which occurs by a mechanism involving oxygen metabolites, has been successfully demonstrated by Weitberg *et al*., 1985 [19]. This is in line with the findings of Sarkar *et al *[13] that confirm the anticlastogenic effect of beta-carotene. Based upon the present study and those reported previously from our laboratory [20] as well as from other laboratories [21] it has been confirmed that **V **at low concentration is not clastogenic in experimental animals. Information on the combined effect of a trace element (**V**) and an antioxidant (BC) in alleviating chemical carcinogenesis in animal model is meager, particularly at molecular level. However, a recent study from our laboratory [22] demonstrate an inhibitory role of concomitant treatment with micronutrient vanadium and VD_3 _on hepatic chromosomal aberrations as well as DNA strand break during rat liver carcinogenesis induced by DENA. In this study we have shown that the occurrence of DDBs are greatly reduced by exploring the inhibitory effect of two antineoplastic agents. **V **and BC thus may be effective in offering protection by possible mechanism by preventing the breaks at DNA by acting on the antiproliferative and differentiation inducing genes.

Histology was taken as end point biomarkers. Histological observations clearly show that DENA in carcinogen control group animals clearly damages the normal architecture of hepatic tissue. A typical vacuolation was observed in the cytoplasm encircling the nucleus with masses of acidophilic materials. Some nuclei in the cells were large and hyperchromatic with prominent and centrally located nucleoli (Figure 3). This condition is known as preneoplastic condition. **V **plus BC supplementation seemed to diminish these changes (Figure 4) and brought back to normal tissue architecture. Mild preneoplastic condition was observed proving that V plus BC synergistically offers chemoprevention and protects against carcinogen-induced damage.

The distribution of trace element levels in various organs indicates a significant differentiation between normal and malignant tissue, which is in good agreement with several previous findings [23]. It is well that malignant cells differ biochemically in many ways from normal cells. Oberley *et al*., 1984 [24] reported that cancer cells have an altered pattern of super oxide dismutase (SOD) activity from that seen in normal cells. They added that cancer cells usually have lowered levels of Cu or Zn-SOD when compared with an appropriate control. Within cells SOD (Mn, Cu, Zn), glutathione peroxidase (Se) and catalase (Fe) remove 0^2- ^and H_2_O_2 _before they approach available promoters of Fenton chemistry [25]. H_2_O_2 _is reduced by glutathione (GSH) [50]. Despite these protective enzymes, some 0^2- ^and H_2_O_2 _may escape and in the presence of decompartmentalized Fe, are catalyzed to more reaction reactive oxygen species (ROS) [25]. This condition is aggravated during carcinogenesis as SOD and GSH concentration reduced during carcinogenic process [26]. In the present study, raised level of Cu or Zn in blood samples of group C, D and especially in group E (which received **V**, BC and **V **plus BC respectively) could be explained by the ability of liver cells to form SOD-model compounds *in vivo*. These complexes scavenge super oxide radicals and account for the antitumor activity of vanadium and beta-carotene in rat hepatic neoplasia presumably by stabilizing the genome.

Several studies have shown augmentation of tumor growth in rodent models with Fe supplementation [27,28]. *In vitro *and *in vivo *data suggest that Fe may be capable of mutagenic effects mediated through free radical generation or tumor promotion through nutritional mechanisms [29]. As a transition metal Fe is possessed of loosely bound electrons that are capable of participating in lipid peroxidation (LPO) reactions. Such reactions are thought to lead to DNA damage and ultimately neoplasia [30]. This is in accordance with observations made in the present study. In this regard, marked lowering in Fe concentration by combined supplementation of **V **and BC to DEN-induced animals in Group E (Table 1) could be explained by the suggested mechanism(s). Treatment with **V **or BC alone or in combination showed decrease in the level of K, Mn, Se concentrations when compare with carcinogenesis control group (group B). At present it is doubtful that minor changes in mineral levels induced by **V **and BC combination played a major role in tumorigenesis in this experiment, but the possibility cannot be excluded.

## Conclusion

These studies suggest that trace element concentration *in vivo *could be used as an early biomarker for carcinogenic process. The results documented indicate that vanadium plus beta-carotene at a given dose can modulate other trace element concentration in serum by some unknown mechanism that is yet to be elucidated and in so doing preserve the genetic stability. The study remains noteworthy in the aspect that it records **V **and BC both-mediated suppression of DNA-strand break at the initiation stage in addition to essential / trace elemental concentrations alteration(s) in the liver and in the maintenance of the normal hepatic architecture during the preneoplastic stage of liver carcinogenesis.

## Authors' contributions

Author 1 MBC carried out the rat hepatocarcinogenesis, morphology, morphometry and histopathological studies. Author 1 MBC and author 3 IK made DNA-chain break studies. Proton induced X-ray emission analysis and assessment was made by author 2 SM and author 4 VV and author 5 MD. Author 6 NBK, a senior medical specialist has done the expert consultant for the pathological aspects of the work. The planning, design and coordination of the experimental work was made by author 7 MC, the principal investigator.
